# Analysis of the clinical characteristics of patients with COVID-19-related deaths during the two epidemic periods in a tertiary hospital in Wuhan, China: A retrospective study

**DOI:** 10.1097/MD.0000000000049397

**Published:** 2026-06-26

**Authors:** Jing-mei Ding, Min Li, Meng-yu Shen, Lei Han, Jin-bing Du

**Affiliations:** a Department of Outpatient, General Hospital of Central Theater Command, Wuhan, Hubei, China; b Department of Medical Laboratory Center, General Hospital of Central Theater Command, Wuhan, Hubei, China; c Department of Medical Administration, General Hospital of Central Theater Command, Wuhan, Hubei, China.

**Keywords:** Alpha strains, clinical features, early death, epidemic period, Omicron strains, patients with COVID-19

## Abstract

The coronavirus disease 2019 (COVID-19) pandemic has triggered a global health crisis, resulting in millions of deaths and exacerbating the burden on healthcare systems. This study analyzed the clinical characteristics of hospitalized COVID-19 patients who died during 2 epidemic periods dominated by the Alpha and Omicron variants in a tertiary hospital in Wuhan, to provide evidence for understanding the effects of different variants of the novel coronavirus and the factors contributing to early death. A retrospective analysis was conducted on 164 COVID-19 in-hospital death cases from January to April 2020 and October 2022 to January 2023. Patients were grouped by epidemic period and death status (early death was defined as death within 28 days after symptom onset), with clinical features compared and early death risk factors explored. Among 164 patients, the mean age was 85.76 years, the male-to-female ratio was 3.69:1, and 152 (92.68%) had comorbidities (hypertension, coronary heart disease, diabetes, cerebrovascular disease, chronic renal insufficiency). The median time from onset to admission was 5.00 days, hospital stay was 12.50 days, and onset to death was 18.00 days; 134 (81.71%) died early. Significant differences (*P* < .05) were found in 16 indicators between epidemic periods and in 29 variables between early versus non-early death groups. Multivariable logistic regression identified 5 independent predictors of early death: second epidemic period (odds ratio [OR] = 44.76), coronary heart disease (OR = 38.96), cerebrovascular disease (OR = 24.65), blood urea nitrogen peak > 18 mmol/L (OR = 23.58), and continuous lactate dehydrogenase (LDH) increase (OR = 9.86). Four protective factors were identified: new-onset anemia (OR = 0.01), lymphocyte (LYM) valley 0.5 to 0.8 × 10^9^/L (OR = 0.02), LDH peak 250 to 400 U/L (OR = 0.01), and hemoglobin (Hb) valley 60 to 90 g/L (OR = 0.01). Hospitalized COVID-19 patients who died early were mainly elderly males with comorbidities. Dynamic changes in key biochemical parameters, particularly blood urea nitrogen, LDH, Hb, and LYM count, demonstrated strong predictive value for early mortality. Elevated biochemical markers indicative of uncontrolled systemic inflammation or progressive organ dysfunction were strongly associated with an increased risk of early mortality. In contrast, a moderated, non-exhaustive physiological response, characterized by preserved LYM counts, stable Hb levels, and non-catastrophic LDH elevation, was independently associated with reduced early death risk, suggesting that functional resilience, rather than maximal immune activation or profound depletion, is protective.

## 1. Introduction

On May 5, 2023, the World Health Organization (WHO) declared that coronavirus disease 2019 (COVID-19) no longer constituted a Public Health Emergency of International Concern, signifying the conclusion of the 3-year-long pandemic emergency and the tragic loss of over 6.9 million lives worldwide. The mortality rate of hospitalized patients with COVID-19 varied significantly across countries, ranging from 20.3% to 21.7% in the United States,^[1]^ 24% in Europe,^[2]^ and higher rates in Spain (42.4%)^[3]^ and Italy (44.3%).^[4]^ China experienced the highest mortality rate during the pandemic, including 2 periods: the initial phase of the COVID-19 pandemic and the early implementation of China’s “Class II Management of Novel Coronavirus” policy by the government, with prevalent virus strains being Alpha and Omicron variants, respectively. Among them, patients infected with the former had a higher rate of severe illness and mortality, often accompanied by severe respiratory damage and systemic inflammatory responses. In contrast, most of the hospitalized patients infected with the latter were mild cases, with severe cases mainly concentrated among the elderly or those with underlying diseases, mainly presenting as upper respiratory symptoms combined with the aggravation of underlying diseases. To date, the WHO has confirmed 5 mutations, including Alpha, Beta, Gamma, Delta, and Omicron variants, each demonstrating enhanced transmission capabilities compared with its predecessor.^[5]^ Given that the transmissibility of the Omicron variant may be up to 10 times higher than that of the original COVID-19 strain and 2 times higher than that of the Delta variant, it has garnered widespread attention.^[6]^ However, most comparative studies on clinical characteristics have focused on comparing Omicron with Delta strain patients,^[7,8]^ with fewer comparisons between Omicron strain patients and those infected with the Alpha strain. Therefore, the objective of this study was to compare the clinical characteristics of patients who died during different epidemic periods, with the primary strains being Alpha and Omicron, in order to enhance our understanding of the impact of different mutant strains of the virus.

## 2. Materials and methods

### 2.1. Data resources

Data on patients with COVID-19-related deaths were collected from discharge records from a tertiary hospital in Wuhan from January 1, 2020, to April 30, 2020 (the first period of the epidemic), and October 1, 2022, to January 31, 2023 (the second period of the epidemic). The tertiary hospital in Wuhan is the General Hospital of Central Theater Command, which is the largest military medical facility in central China and is also the first designated medical treatment institution after the outbreak of the novel coronavirus in Wuhan. There were 164 patients with confirmed novel coronavirus infection who were admitted to the hospital and ultimately died (the criterion for confirming novel coronavirus infection was a positive nucleic acid test within 24 hours before or after admission), including basic information and hospitalization conditions of the patients, such as admission time, age, sex, and length of stay (LOS), and results of many kinds of clinical tests during hospitalization, including inflammatory factors, cytokines, lymphocyte count, troponin T, and more than 30 other indicators. If there was missing data for a single patient on a single indicator, the sample average would be used to replace it. If the number of missing patients for a particular indicator exceeded 3, that indicator would not be included in the statistical analysis process.

### 2.2. Statistical analysis

The statistical analysis process used in this study can be divided into 3 steps. The first step was the statistical description, which described the distribution of the relevant variables, the mean ± standard deviation for normal variables, the median (Q1–Q3) for non-normal variables, and the confidence intervals estimated for all relevant parameters. The second step was the univariate analysis, which only considered 1 variable when comparing the differences among groups; that is, exploring whether there were any differences among groups in only 1 aspect. Common methods included analysis of variance for variables with a normal distribution, Mann–Whitney *U* tests, and Kruskal–Wallis *H (K*) tests for variables with a non-normal distribution. In this study, the grouping criteria were “the epidemic period in which the cases occurred” and “whether patients experienced early death (death occurred within 28 days after the onset of symptoms).” The third step was a binary logistic regression to explore the probable influencing factors of early COVID-19-related deaths. The dependent variable was “whether the case experiences early death,” and the independent variables were various baseline information and clinical laboratory indicators that show statistically significant differences between early death and non-early death in the previous statistical analysis, where the peak and valley values of the corresponding indicators were stratified based on clinical significance, with normal range values set as the control group and other abnormal value ranges set as different dummy variables, and all dummy variables were included in the independent variables for regression analysis (for detailed dummy variable coding, please refer to the Supplementary Table, Supplemental Digital Content 1). Statistical analyses were performed using IBM SPSS Statistics 23.0. Significance testing was two-sided, and the significance threshold was set at *P* < .05.

### 2.3. Ethical approval

Not applicable. All data were collected by the Department of Information from the hospital management system and did not contain any private information on the study subjects.

## 3. Results

### 3.1. Basic information of patients with COVID-19-related death during the 2 epidemic periods

In a cohort of 164 fatalities associated with COVID-19, the distribution of deaths across the epidemic waves was characterized by 23 (14.02%) during the first period (January 1, 2020, to April 30, 2020) and 141 (85.98%) during the second period (October 1, 2022, to January 31, 2023). The majority of cases were male, with 129 individuals (78.66%) compared with 35 females (21.34%). The average age of all cases was 85.76 (74.98, 92.67) years. Notably, 160 patients (97.56%) were above 60 years of age. Stratification based on the WHO’s age categorization for the elderly, designated as young-old (60–74 years), old-old (75–89 years), and oldest-old (90 years and above), demonstrated that the old-old group predominated with 63 cases (38.41%), closely followed by the oldest-old group with 61 cases (37.20%), and the young-old group with 36 cases (21.95%). Among the 164 patients, 131 (79.88%) had no smoking history, and 137 (83.54%) had no history of alcohol consumption. The most prevalent comorbidities accompanying these patients were hypertension (114 cases, 69.51%), coronary atherosclerotic heart disease (60 cases, 36.59%), diabetes (53 cases, 32.32%), cerebrovascular diseases (52 cases, 31.71%), and chronic kidney disease (45 cases, 27.44%). A substantial proportion of the patients, 94 (57.32%), presented with 3 or more concurrent underlying conditions. Comparative analysis of the demographic and clinical characteristics of COVID-19-related deaths, categorized by the period of the epidemic, revealed a statistically significant difference in the prevalence of “comorbidities” (χ^2^ = 9.545, *P* = .02), which indicated that cases in the second epidemic period were significantly associated with a higher number of comorbidities. For more information, please refer to Table 1.

**Table 1:** Basic information of patients with COVID-related deaths (n [%] or M [Q1–Q3]).

	Total (n = 164)	The first epidemic period (n = 23)	The second epidemic period (n = 141)	Statistics	*P* value
Gender					
Male	129 (78.66)	18 (78.26)	111 (78.72)	χ^2^ = 0.00	.96
Female	35 (21.34)	5 (21.74)	30 (21.28)		
Age	85.76 (74.98, 92.67)	78.12 (71.09, 90.35)	86.50 (76.00, 93.04)	*Z* = −1.65	.10
19–44 yr	3 (1.83)	2 (8.70)	1 (0.71)	χ^2^ = 7.69	.10
45–59 yr	1 (0.61)	0	1 (0.71)		
60–74 yr	36 (21.95)	6 (26.09)	30 (21.28)		
75–89 yr	63 (38.41)	8 (34.78)	55 (39.01)		
≥90 yr	61 (37.20)	7 (30.43)	54 (38.30)		
Smoking	33 (20.12)	4 (17.39)	29 (20.57)	*Z* = 0.22	.90
Alcohol	27 (16.46)	3 (13.04)	24 (17.02)	*Z* = 0.24	.88
Comorbidities					
Hypertension	114 (69.51)	13 (56.52)	101 (71.63)	*Z* = 2.13	.14
Coronary heart disease	60 (36.59)	10 (43.48)	50 (35.46)	*Z* = 0.55	.46
Diabetes	53 (32.32)	4 (17.39)	49 (34.75)	*Z* = 2.73	.10
Cerebrovascular disease	52 (31.71)	4 (17.39)	48 (34.04)	*Z* = 2.53	.11
Chronic renal insufficiency	45 (27.44)	4 (17.39)	41 (29.08)	*Z* = 1.36	.24
Malignancy	27 (16.46)	2 (8.70)	25 (17.73)	*Z* = 1.17	.28
Hyperlipidemia	14 (8.54)	1 (4.35)	13 (9.22)	*Z* = 0.60	.44
Chronic obstructive pulmonary disease	9 (5.49)	0	9 (6.38)	*Z* = 1.55	.21
Senile dementia	9 (5.49)	1 (4.35)	8 (5.67)	*Z* = 0.07	.80
Bronchiectasis	6 (3.66)	0	6 (4.26)	*Z* = 1.02	.31
Other diseases	22 (13.41)	2 (8.70)	20 (14.18)	*Z* = 0.51	.47
Number of comorbidities					
0	12 (7.32)	4 (17.39)	8 (5.67)	χ^2^ = 9.545	.02*
1	25 (15.24)	1 (4.35)	24 (17.02)		
2	33 (20.12)	8 (34.78)	25 (17.73)		
3 or more	94 (57.32)	10 (43.48)	84 (59.57)		

* *P* < .05, the difference in “Number of comorbidities” of patients between the 2 epidemic periods was statistically significant, which indicated that the patients from the second period had a higher number of comorbidities.

### 3.2. Clinical characteristics of patients with COVID-19-related death during the 2 epidemic periods

In this study, we analyzed 164 COVID-19-related fatalities. The median time from symptom onset to hospital admission was 5.00 (1.00, 9.00) days. The median LOS was 12.50 (6.00, 19.75) days. Furthermore, the median time from symptom onset to death was 18.00 (13.00, 25.00) days. Notably, 134 patients (81.71%) died within 28 days post-symptom onset, and 147 patients (89.63%) died within 28 days of hospital admission. Among the deceased, 114 patients (69.51%) reported fever as a preadmission symptom, with a peak body temperature of 38.50 (28.00, 39.00) °C; 43 cases (26.22%) exhibited respiratory distress upon admission, while 16 cases (9.76%) presented with altered consciousness. Analyzing the direct causes of death, multiple organ failure was the leading cause, accounting for 77 cases (46.95%), followed by respiratory failure in 71 cases (43.29%). During their hospital stay, the most frequently used respiratory support measures among the 164 cases were continuous oxygen therapy in 59 cases (35.98%), invasive mechanical ventilation in 54 cases (32.93%), and continuous high-flow oxygen therapy in 23 cases (14.02%).

In our analysis of 164 hospitalized patients, we observed the following clinical biomarker trends: the peak C-reactive protein (CRP) levels reached 125.53 ± 79.41 mg/L, and a continuous increase in CRP levels was noted in 96 cases (58.54%); procalcitonin (PCT) peaked at 1.72 (0.58, 7.61) ng/mL, and 109 cases (66.46%) exhibited a sustained rise; interleukin-6 (IL-6) levels peaked at 253.40 (115.50, 679.80) pg/mL, and 98 cases (59.76%) showed a continuous increase; the absolute nadir count of lymphocytes (LYM) was recorded at 0.32 (0.19, 0.54) × 10^9^/L, and 112 cases (68.29%) experienced a continuous decline; hyperkalemia was observed in 63 cases (38.41%); myoglobin (MYO) levels peaked at a median of 563.70 (235.80, 1523.00), and 100 cases (60.98%) showed a continuous increase; cardiac troponin T (cTnT) peaked at 0.14 (0.07, 0.35) ng/mL, and 118 cases (71.95%) had a continuous increase; amino-terminal pro-brain natriuretic peptide (N-pro BNP) peaked at a median of 6986.00 (2813.00, 16,157.00) pg/mL, and 111 cases (67.68%) displayed a continuous increase; creatine kinase-MB (CK-MB) levels peaked at 5.91 (3.02, 13.09) ng/mL, and 97 cases (59.15%) had a continuous increase; the nadir value of albumin was 27.60 (25.30, 29.65) g/L, and 109 cases (66.46%) showed a continuous decrease; lactate dehydrogenase (LDH) peaked at 533.00 (376.50, 761.50) U/L, and 100 cases (60.98%) had a continuous increase; serum creatinine (Scr) levels peaked at 169.50 (94.25, 332.50) µmol/L, and 92 cases (56.10%) showed a continuous increase; blood urea nitrogen (BUN) levels peaked at 21.30 (12.96, 32.81) mmol/L, and 111 cases (67.68%) had a continuous increase; activated partial thromboplastin time (APTT) peaked at 34.40 (31.40, 40.50) seconds, and 81 cases (49.39%) showed a continuous increase; prothrombin time (PT) peaked at 14.00 (12.10, 16.80) seconds, and 92 cases (56.10%) had a continuous increase; D-dimer (D-D) levels peaked at 3.33 (1.11, 9.87) mg/L, and 90 cases (54.88%) showed a continuous increase; hemoglobin (Hb) levels reached a nadir of 95.53 ± 23.93 g/L, and 127 cases (77.44%) showed a continuous decrease; 45 cases (27.44%) developed new-onset anemia; platelet (PLT) levels reached a nadir of 86.00 (54.00, 132.00) × 10^9^/L, and 76 cases (46.34%) showed a continuous decrease; and the peak value of red cell distribution width (RDW) was 15.30% (13.90%, 16.50%). Notably, aside from the APTT and PT, which did not differ significantly from the upper limits of the normal population, all other biomarkers exhibited peak values significantly above the normal range. Conversely, the nadir values for lymphocyte count, albumin, Hb, and PLT were significantly below the normal range.

Further comparative analysis of the patients’ clinical characteristics between the 2 epidemic periods revealed statistically significant differences across the 15 clinical indicators. These indicators included the time from symptom onset to hospital admission, the interval from symptom onset to death, the mortality rate within 28 days post-symptom onset, the prevalence of respiratory distress at the time of admission, the respiratory support interventions implemented, and trends in biomarker levels during hospitalization. Specifically, the clinical biomarkers and their trends with significant differences were as follows: the number of cases with a continuous increase in CRP, PCT, and CK-MB levels; the peak values of CK-MB, LDH, APTT, PT, D-D, and RDW; and the number of cases with a continuous decrease in PLT levels. All of these differences were statistically significant (*P* < .05). Additional details are provided in Table 2.

**Table 2:** Clinical characteristics of patients with COVID-related deaths (n [%] or M [Q1–Q3]).

	Total (n = 164)	The first epidemic period (n = 23)	The second epidemic period (n = 141)	Statistics	*P* value
Time from symptom to admission (d)	5.00 (1.00, 9.00)	8.00 (3.00, 10.00)	4.00 (0, 8.50)	*Z* = −2.60	.01*
LOS (d)	12.50 (6.00, 19.75)	16.00 (7.00, 28.00)	12.00 (5.50, 19.00)	*Z* = −1.31	.19
Time from symptom to death (d)	18.00 (13.00, 25.00)	21.00 (16.00, 34.00)	18.00 (12.75, 23.50)	*Z* = −2.55	.01*
Death within 28 days from symptom (n)	134 (81.71)	14 (60.87)	120 (85.11)	χ^2^ = 7.77	.01*
Death within 28 days from admission (n)	147 (89.63)	18 (78.26)	129 (91.49)	χ^2^ = 3.72	.05
Preadmission symptoms					
Fever (n)	114 (69.51)	18 (78.26)	96 (68.09)	χ^2^ = 1.00	.33
Peak of fever (°C)	38.50 (38.00, 39.00)	38.10 (37.95, 39.00)	38.50 (38.00, 39.00)	*Z* = −0.78	.43
Dyspnea (n)	43 (26.22)	0	43 (30.50)	χ^2^ = 9.51	<.01*
Unconsciousness (n)	16 (9.76)	0	16 (11.35)	χ^2^ = 2.89	.09
Direct cause of death					
Respiratory failure (n)	71 (43.29)	7 (30.43)	64 (45.39)	χ^2^ = 1.80	.18
Acute myocardial injury (n)	10 (6.10)	0	10 (7.09)	χ^2^ = 1.74	.19
Multiple organ failure (n)	77 (46.95)	15 (65.22)	62 (43.97)	χ^2^ = 3.58	.06
Other Causes (n)	6 (3.66)	1 (4.35)	5 (3.55)	χ^2^ = 0.04	.09
Respiratory support					
None (n)	6 (3.66)	0	6 (4.26)	χ^2^ = 73.88	<.001*
Continuous oxygen therapy (n)	59 (35.98)	5 (21.74)	59 (35.98)		
Continuous high-flow oxygen therapy (n)	23 (14.02)	0	23 (14.02)		
Noninvasive ventilator (n)	22 (13.41)	16 (69.57)	22 (13.41)		
Invasive ventilator (n)	54 (32.93)	2 (8.70)	54 (32.93)		
Peak value of CRP (mg/L)	125.53 ± 79.41	126.85 ± 85.93	122.94 ± 78.52	*Z* = 0.05	.83
CRP with continuous increase (n)	96 (58.54)	18 (78.26)	78 (55.32)	χ^2^ = 4.29	.04*
Peak value of PCT (ng/mL)	1.72 (0.58, 7.61)	0.97 (0.40, 10.00)	1.72 (0.61, 7.40)	*Z* = −0.47	.64
PCT with continuous increase (n)	109 (66.46)	20 (86.96)	89 (63.12)	χ^2^ = 5.04	.03*
Peak value of IL-6 (pg/mL)	253.40 (115.50, 679.80)	371.90 (87.20, 757.10)	252.20 (116.70, 666.98)	*Z* = −0.14	.89
IL-6 with continuous increase (n)	98 (59.76)	18 (78.26)	80 (56.74)	χ^2^ = 3.81	.05
Valley value of LYM (10^9^/L)	0.32 (0.19, 0.54)	0.26 (0.16, 0.43)	0.32 (0.19, 0.56)	*Z* = −1.10	.27
LYM with continuous decrease (n)	112 (68.29)	15 (65.22)	97 (68.79)	χ^2^ = 0.12	.73
Hyperkalemia occurrence (n)	63 (38.41)	54 (38.30)	9 (39.13)	χ^2^ = 0.01	.94
Peak value of MYO (ng/mL)	563.70 (235.80, 1523.00)	843.10 (220.60, 2003.00)	538.20 (235.90, 1484.00)	*Z* = −0.97	.33
MYO with continuous increase (n)	100 (60.98)	17 (73.91)	83 (58.87)	χ^2^ = 1.88	.17
Peak value of cTnT (ng/mL)	0.14 (0.07, 0.35)	0.32 (0.05, 0.091)	0.13 (0.07, 0.29)	*Z* = −1.17	.24
cTnT with continuous increase (n)	118 (71.95)	18 (78.26)	100 (70.92)	χ^2^ = 0.53	.47
Peak value of N-pro BNP (pg/mL)	6986.00 (2813.00, 16,157.00)	5204.00 (2026.00, 8808.00)	7354.00 (2853.25, 17,630.00)	*Z* = −1.08	.28
N-pro BNP with continuous increase (n)	111 (67.68)	19 (82.61)	92 (65.25)	χ^2^ = 2.73	.10
Peak value of CK-MB (ng/mL)	5.91 (3.02, 13.09)	13.82 (5.45, 28.45)	5.71 (2.93, 10.69)	*Z* = −2.70	<.01*
CK-MB with continuous increase (n)	97 (59.15)	18 (78.26)	79 (56.03)	χ^2^ = 4.05	.04*
Valley value of albumin (g/L)	27.60 (25.30, 29.65)	26.40 (24.10, 28.40)	27.65 (25.48, 29.70)	*Z* = −1.64	.10
Albumin with continuous decrease (n)	109 (66.46)	13 (56.52)	96 (68.09)	χ^2^ = 1.19	.28
Peak value of LDH (U/L)	533.00 (376.50, 761.50)	711.00 (500.00, 1879.00)	505.50 (351.50, 692.25)	*Z* = −2.79	<.01*
LDH with continuous increase (n)	100 (60.98)	8 (65.22)	85 (60.28)	χ^2^ = 0.20	.65
Peak value of Scr (µmol/L)	169.50 (94.25, 332.50)	182.00 (83.00, 270.00)	167.00 (94.50, 337.00)	*Z* = −0.54	.59
Scr with continuous increase (n)	92 (56.10)	14 (60.87)	78 (55.32)	χ^2^ = 0.25	.62
Peak value of BUN (mmol/L)	21.30 (12.96, 32.81)	18.70 (14.26, 34.44)	21.50 (12.75, 32.52)	*Z* = −0.05	.96
BUN with continuous increase (n)	111 (67.68)	17 (73.91)	47 (66.67)	χ^2^ = 0.48	.49
Peak value of APTT (s)	34.40 (31.40, 40.50)	41.90 (32.90, 68.80)	33.70 (31.03, 39.15)	*Z* = −2.99	<.01*
APTT with continuous increase (n)	81 (49.39)	14 (60.87)	67 (47.52)	χ^2^ = 1.41	.24
Peak value of PT (s)	14.00 (12.10, 16.80)	16.90 (15.10, 30.20)	13.60 (12.00, 15.80)	*Z* = −3.55	<.001*
PT with continuous increase (n)	92 (56.10)	17 (73.91)	75 (53.19)	χ^2^ = 3.45	.06
Peak value of D-D (mg/L)	3.33 (1.11, 9.87)	5.23 (2.82, 13.12)	2.98 (0.95, 9.19)	*Z* = −2.36	.02*
D-D with continuous increase (n)	90 (54.88)	75 (53.19)	15 (65.22)	χ^2^ = 1.16	.28
Valley value of Hb (g/L)	95.53 ± 23.93	87.20 ± 26.95	96.89 ± 23.25	*Z* = 2.86	.09
Hb with continuous decrease (n)	127 (77.44)	18 (78.26)	109 (77.30)	χ^2^ = 0.01	.92
New-onset anemia during hospitalization (n)	45 (27.44)	9 (39.13)	36 (25.53)	χ^2^ = 1.84	.18
Valley value of PLT (10^9^/L)	86.00 (54.00, 132.00)	63.00 (30.75, 100.50)	91.00 (55.00, 135.00)	*Z* = −1.44	.15
PLT with continuous decrease (n)	76 (46.34)	15 (65.22)	61 (43.26)	χ^2^ = 3.83	.04*
Peak value of RDW (%)	15.30 (13.90, 16.50)	16.10 (15.40, 17.20)	15.20 (13.80, 16.35)	*Z* = −2.85	<.01*

The data in the table showed that, compared with patients in the first epidemic period, those in the second epidemic period had a shorter “time from symptoms to admission (4.00 vs 8.00, *P* = .01),” a shorter “time from symptoms to death (18.00 vs 21.00, *P* = .01),” a higher proportion of patients who died within 28 days of symptoms (85.11% vs 60.87%, *P* = .01), a higher proportion of patients who had dyspnea before admission (30.50% vs 0, *P* < .01), and a more significant difference in the respiratory support used during hospitalization (*P* < .001). Among multiple biological indicators, the peak values of CK-MB, LDH, APTT, PT, D-D, and RDW were lower in patients in the second epidemic period (5.71 vs 13.82, *P* < .01; 505.50 vs 711.00, *P* < .01; 33.70 vs 41.90, *P* < .01; 13.60 vs 16.90, *P* < .001; 2.98 vs 5.23, *P* = .02; and 15.20% vs 16.10%, *P* < .01, respectively). In terms of the continuous change of biological indicators, the proportion of patients in the second epidemic period whose CRP continued to increase (55.32% vs 78.26%, *P* = .04), PCT continued to increase (63.12% vs 86.96%, *P* = .03), CK-MB continued to increase (56.03% vs 78.26%, *P* = .04), and PLT continued to decrease (43.26% vs 65.22%, *P* = .04) was lower.

APTT = activated partial thromboplastin time, BUN = blood urea nitrogen, CK-MB = creatine kinase-MB, CRP = C-reactive protein, cTnT = cardiac troponin T, D-D = D-dimer, Hb = hemoglobin, IL-6 = interleukin-6, LDH = lactate dehydrogenase, LOS = length of stay, LYM = lymphocytes, MYO = myoglobin, N-pro BNP = amino-terminal pro-brain natriuretic peptide, PCT = procalcitonin, PLT = platelets, PT = prothrombin time, RDW = red cell distribution width, Scr = serum creatinine.

* *P* < .05, the difference between patients in the 2 epidemic periods was statistically significant.

### 3.3. Comparison of basic and clinical information between early and non-early death

In this study, patients who died within 28 days post-symptom onset were categorized as “early death” patients, while those who died after this period were classified as “non-early death” patients. A total of 134 patients (81.70%) were identified as having experienced early death, with an average age of 83.95 (75.59, 91.13) years old. The majority of these patients were within the age brackets of “90 years and older” and “75 to 89 years,” accounting for a cumulative proportion of 75.37%. Among them, 30 (22.39%) reported a history of smoking, and 25 (18.66%) reported a history of alcohol consumption. The most prevalent comorbidity among early death patients was hypertension, affecting 94 cases (70.15%), followed by coronary atherosclerotic heart disease (55 cases, 41.04%), cerebrovascular disease (48 cases, 35.82%), diabetes (44 cases, 32.84%), and chronic kidney disease (43 cases, 32.09%). Notably, 81 patients (60.45%) had 3 or more comorbid conditions. Upon statistical comparison of basic information between early and non-early death patients, significant differences were observed in the prevalence of coronary atherosclerotic heart disease, cerebrovascular diseases, chronic kidney disease, and the number of comorbid conditions (*P* < .05). For more detailed information, refer to Table 3.

**Table 3:** Comparison of the basic information of patients with early and non-early death (n [%] or M [Q1–Q3]).

	Total (n = 164)	Early death (n = 134)	Non-early death (n = 30)	Statistics	*P* value
Gender					
Male	129 (78.66)	109 (81.34)	20 (66.67)	χ^2^ = 3.15	.08
Female	35 (21.34)	25 (18.66)	10 (33.33)		
Age	85.76 (74.98, 92.67)	83.95 (75.59, 91.13)	86.57 (74.54, 93.04)	*Z* = −0.95	.34
19–44 yr	3 (1.83)	3 (1.83)	0	χ^2^ = 3.31	.51
45–59 yr	1 (0.61)	1 (0.61)	0		
60–74 yr	36 (21.95)	29 (21.64)	7 (23.33)		
75–89 yr	63 (38.41)	48 (35.82)	15 (50.00)		
≥90 yr	61 (37.20)	53 (39.55)	8 (26.67)		
Smoking	33 (20.12)	30 (22.39)	3 (10.00)	χ^2^ = 2.34	.13
Alcohol	27 (16.46)	25 (18.66)	2 (6.67)	χ^2^ = 2.56	.11
Comorbidities					
Hypertension	114 (69.51)	94 (70.15)	20 (66.67)	χ^2^ = 0.14	.71
Coronary heart disease	60 (36.59)	55 (41.04)	5 (16.67)	χ^2^ = 6.28	.01*
Diabetes	53 (32.32)	44 (32.84)	9 (30.00)	χ^2^ = 0.09	.76
Cerebrovascular disease	52 (31.71)	48 (35.82)	4 (13.33)	χ^2^ = 5.72	.02*
Chronic renal insufficiency	45 (27.44)	43 (32.09)	2 (6.67)	χ^2^ = 7.96	<.01*
Malignancy	27 (16.46)	23 (17.16)	4 (13.33)	χ^2^ = 0.26	.61
Hyperlipidemia	14 (8.54)	11 (8.21)	3 (10.00)	χ^2^ = 0.10	.75
Chronic obstructive pulmonary disease	9 (5.49)	9 (6.72)	0	χ^2^ = 2.13	.14
Senile dementia	9 (5.49)	9 (6.72)	0	χ^2^ = 2.31	.14
Bronchiectasis	6 (3.66)	5 (3.73)	1 (3.33)	χ^2^ = 0.01	.92
Other diseases	22 (13.41)	16 (11.94)	6 (20.00)	χ^2^ = 1.37	.24
Number of comorbidities					
0	12 (7.32)	6 (4.48)	6 (20.00)	χ^2^ = 10.09	.02*
1	25 (15.24)	19 (14.18)	6 (20.00)		
2	33 (20.12)	28 (20.90)	5 (16.67)		
3 or more	94 (57.32)	81 (60.45)	13 (43.33)		

The data in the table show that, compared with non-early death patients, the proportion of patients with coronary heart disease, cerebrovascular disease, and chronic renal insufficiency was higher in early death patients (41.04% vs 16.67%, *P* = .01; 35.82% vs 13.33%, *P* = .02; 32.09% vs 6.67%, *P* < .01), and the proportion of patients with multiple comorbidities was higher in early death patients than in non-early death patients (*P* = .02).

* *P* < .05, the differences between patients in the 2 epidemic periods were statistically significant.

Further comparison of the clinical characteristics between early and non-early death patients revealed significant differences. Early death patients had significantly shorter “time from symptom to admission (*P* < .001)” and “LOS (*P* < .001)” than non-early death. The proportion of patients with “multiple organ failure” among early death patients was significantly lower than that among non-early death patients (43.28% vs 63.33%, *P* < .001). Among the remaining 37 clinical biomarkers, there were statistically significant differences in 22 indicators, of which 5 peak values were significantly lower in patients with early death than in patients with non-early death, including “CRP (113.25 ± 75.52 vs 166.79 ± 82.07, *P* < .01),” “IL-6 (239.00 vs 506.50, *P* = .04),” “albumin (34.67 ± 4.22 vs 39.16 ± 5.22, *P* < .001),” “PT (13.60 vs 15.80, *P* < .01)” and “RDW (15.25% vs 16.50%, *P* < .01),” while there were 3 valley values were much higher in patients with early death than in patients with non-early death, including “LYM count (0.33 vs 0.25, *P* = .03),” “Hb (100.07 ± 22.96 vs 78.43 ± 19.67, *P* < .001)” and “PLT (91.00 × 10^9^/L vs 70.50 × 10^9^/L, *P* = .04).” Among the remaining 14 indicators of disease severity, significant differences were observed between the patients who experienced early death and those who did not. Notably, the proportion of patients with a continuous increase or decrease in certain biomarkers during hospitalization was markedly lower in the early death group than that in the non-early death group. The specific indicators with significant differences include: “continuous increase in CRP (54.48% vs 76.67%, *P* = .03),” “continuous increase in PCT (61.19% vs 90.00%, *P* < .01),” “continuous increase in IL-6 (54.48% vs 83.33%, *P* < .01),” “continuous increase in MYO (53.73% vs 93.33%, *P* < .001),” “continuous increase in cTnT (66.42% vs 96.67%, *P* < .01),” “continuous increase in N-pro BNP (62.69% vs 90.00%, *P* < .01),” “continuous increase in CK-MB (54.48% vs 80.00%, *P* = .01),” “continuous increase in Scr (52.24% vs 73.33%, *P* = .04),” “continuous increase in BUN (64.18% vs 83.33%, *P* = .04),” “continuous increase in APTT (43.28% vs 76.67%, *P* < .01),” “continuous increase in PT (50.75% vs 80.00%, *P* = .03),” “continuous decrease in Hb (72.39% vs 100.00%, *P* < .01),” “continuous decrease in PLT (41.79% vs 66.67%, *P* = .01),” and “new-onset anemia during hospitalization (19.40% vs 63.33%, *P* < .001).” These findings underscore the association between the severity of biomarker trends and risk of early death in patients. For further details, please refer to Table 4.

**Table 4:** Comparison of clinical characteristics of patients with early and non-early death (n [%] or M [Q1–Q3]).

	Total (n = 164)	Early death (n = 134)	Non-early death (n = 30)	Statistics	*P* value
Time from symptom to admission (d)	5.00 (1.00, 9.00)	4.00 (0.88, 8.00)	7.50 (3.00, 12.25)	*Z* = −2.25	.02*
LOS (d)	12.50 (6.00, 19.75)	10.00 (5.00, 16.00)	29.00 (21.00, 36.00)	*Z* = −7.33	<.001*
Preadmission symptoms					
Fever (n)	114 (69.51)	91 (67.91)	23 (76.67)	χ^2^ = 0.89	.35
Peak of fever (°C)	38.50 (38.00, 39.00)	38.50 (38.00, 39.00)	38.50 (37.60, 39.00)	*Z* = −0.44	.66
Dyspnea (n)	43 (26.22)	37 (27.61)	6 (20.00)	χ^2^ = 0.73	.39
Unconsciousness (n)	16 (9.76)	14 (10.45)	2 (6.67)	χ^2^ = 0.40	.53
Direct cause of death					
Respiratory failure (n)	71 (43.29)	62 (46.27)	9 (20.00)	χ^2^ = 2.64	.10
Acute myocardial injury (n)	10 (6.10)	9 (6.72)	1 (3.33)	χ^2^ = 0.49	.48
Multiple organ failure (n)	77 (46.95)	58 (43.28)	19 (63.33)	χ^2^ = 3.96	.04*
Other Causes (n)	6 (3.66)	5 (3.73)	1 (3.33)	χ^2^ = 0.01	.92
Respiratory support					
None (n)	6 (3.66)	5 (3.73)	1 (3.33)	χ^2^ = 4.71	.32
Continuous oxygen therapy (n)	59 (35.98)	51 (38.06)	0		
Continuous high-flow oxygen therapy (n)	23 (14.02)	21 (15.67)	2 (6.67)		
Noninvasive ventilator (n)	22 (13.41)	16 (11.94)	6 (20.00)		
Invasive ventilator (n)	54 (32.93)	41 (30.60)	13 (43.33)		
Peak value of CRP (mg/L)	125.53 ± 79.41	113.25 ± 75.52	166.79 ± 82.07	*Z* = 11.39	<.01*
CRP with continuous increase (n)	96 (58.54)	73 (54.48)	23 (76.67)	χ^2^ = 4.97	.03*
Peak value of PCT (ng/mL)	1.72 (0.58, 7.61)	1.60 (0.58, 6.07)	2.49 (0.69, 10.20)	*Z* = −0.77	.50
PCT with continuous increase (n)	109 (66.46)	82 (61.19)	27 (90.00)	χ^2^ = 9.13	<.01*
Peak value of IL-6 (pg/mL)	253.40 (115.50, 679.80)	239.00 (110.55, 621.25)	506.50 (151.05, 1407.50)	*Z* = −2.06	.04*
IL-6 with continuous increase (n)	98 (59.76)	73 (54.48)	25 (83.33)	χ^2^ = 8.49	.01*
Valley value of LYM (10^9^/L)	0.32 (0.19, 0.54)	0.33 (0.21, 0.59)	0.25 (0.16, 0.39)	*Z* = −2.17	.03*
LYM with continuous decrease (n)	112 (68.29)	89 (66.42)	23 (76.67)	χ^2^ = 1.19	.28
Hyperkalemia occurrence (n)	63 (38.41)	49 (36.57)	14 (46.67)	χ^2^ = 1.06	.30
Peak value of MYO (ng/mL)	563.70 (235.80, 1523.00)	532.15 (234.63, 1539.25)	753.30 (328.57, 1509.25)	*Z* = −0.54	.59
MYO with continuous increase (n)	100 (60.98)	72 (53.73)	28 (93.33)	χ^2^ = 16.16	<.001*
Peak value of cTnT (ng/mL)	0.14 (0.07, 0.35)	0.13 (0.007, 0.35)	0.16 (0.09, 0.42)	*Z* = −0.50	.62
cTnT with continuous increase (n)	118 (71.95)	89 (66.42)	29 (96.67)	χ^2^ = 11.11	<.01*
Peak value of N-pro BNP (pg/mL)	6986.00 (2813.00, 16,157.00)	6626.00 (2732.00, 16,637.00)	8076.00 (4041.25, 16,269.50)	*Z* = −0.76	.45
N-pro BNP with continuous increase (n)	111 (67.68)	84 (62.69)	27 (90.00)	χ^2^ = 8.36	<.01*
Peak value of CK-MB (ng/mL)	5.91 (3.02, 13.09)	5.83 (2.90, 12.92)	6.49 (4.54, 14.90)	*Z* = −1.10	.27
CK-MB with continuous increase (n)	97 (59.15)	73 (54.48)	24 (80.00)	χ^2^ = 6.61	.01*
Valley value of albumin (g/L)	35.54 ± 4.76	34.67 ± 4.22	39.16 ± 5.22	*Z* = 24.85	<.001*
Albumin with continuous decrease (n)	109 (66.46)	87 (64.93)	22 (73.33)	χ^2^ = 0.78	.38
Peak value of LDH (U/L)	533.00 (376.50, 761.50)	520.50 (383.25, 782.50)	543.50 (311.50, 704.25)	*Z* = −0.16	.88
LDH with continuous increase (n)	100 (60.98)	81 (60.45)	19 (63.33)	χ^2^ = 0.09	.77
Peak value of Scr (µmol/L)	169.50 (94.25, 332.50)	180.00 (95.75, 360.00)	124.50 (80.00, 226.25)	*Z* = −1.93	.05
Scr with continuous increase (n)	92 (56.10)	70 (52.24)	22 (73.33)	χ^2^ = 4.43	.04*
Peak value of BUN (mmol/L)	21.30 (12.96, 32.81)	21.59 (13.59, 32.24)	16.99 (12.27, 34.03)	*Z* = −0.84	.40
BUN with continuous increase (n)	111 (67.68)	86 (64.18)	25 (83.33)	χ^2^ = 4.11	.04*
Peak value of APTT (s)	34.40 (31.40, 40.50)	34.10 (31.40, 39.75)	37.50 (31.30, 48.13)	*Z* = −1.46	.14
APTT with continuous increase (n)	81 (49.39)	58 (43.28)	23 (76.67)	χ^2^ = 10.93	<.01*
Peak value of PT (s)	14.00 (12.10, 16.80)	13.60 (11.88, 15.88)	15.80 (13.65, 25.45)	*Z* = −3.10	<.01*
PT with continuous increase (n)	92 (56.10)	66 (50.75)	24 (80.00)	χ^2^ = 8.52	<.01*
Peak value of D-D (mg/L)	3.33 (1.11, 9.87)	2.98 (0.93, 9.67)	4.62 (1.85, 10.88)	*Z* = −1.45	.15
D-D with continuous increase (n)	90 (54.88)	71 (52.99)	19 (63.33)	χ^2^ = 1.06	.30
Valley value of Hb (g/L)	95.53 ± 23.93	100.07 ± 22.96	78.43 ± 19.67	*Z* = 22.28	<.001*
Hb with continuous decrease (n)	127 (77.44)	97 (72.39)	30 (100.00)	χ^2^ = 10.70	<.01*
New-onset anemia during hospitalization (n)	45 (27.44)	26 (19.40)	19 (63.33)	χ^2^ = 23.76	<.001*
Valley value of PLT (10^9^/L)	86.00 (54.00, 132.00)	91.00 (55.00, 138.50)	70.50 (36.75, 102.50)	*Z* = −2.06	.04*
PLT with continuous decrease (n)	76 (46.34)	56 (41.79)	20 (66.67)	χ^2^ = 6.10	.01*
Peak value of RDW (%)	15.30 (13.90, 16.50)	15.25 (13.80, 16.10)	16.50 (15.10, 17.35)	*Z* = −2.92	<.01*

The data in the table showed that, compared with non-early death patients, early death patients had a shorter “time from symptoms to admission (4.00 vs 7.50, *P* = .02),” significantly shorter “LOS (10.00 vs 29.00, *P* < .001),” and a lower proportion of death due to “multiple organ failure (43.28% vs 63.33%, *P* = .04).” Among the many biological indicators, early death patients had lower peak values of CRP (113.25 ± 75.52 vs 166.79 ± 82.07, *P* < .01), IL-6 (239.00 vs 506.50, *P* = .04), valley values of albumin (34.67 ± 4.22 vs 39.16 ± 5.22, *P* < .001), PT peak values (13.60 vs 15.80, *P* < .01), a lower proportion of new-onset anemia patients during hospitalization (19.40% vs 63.33%, *P* < .001), and a lower peak value of RDW (15.25% vs 16.50, *P* < .01), while early death patients had higher valley values of LYM (0.33 × 10^9^ vs 0.25 × 10^9^, *P* = .03), Hb (100.07 ± 22.96 vs 78.43 ± 19.67, *P* < .001), and PLT (91.00 vs 70.50, *P* = .04) than non-early death patients. In terms of the continuous change of biological indicators, the proportion of early death patients with continuously increasing CRP (54.48% vs 76.67%, *P* = .03), PCT (61.19% vs 90.00%, *P* < .01), IL-6 (54.48% vs 83.33%, *P* = .01), MYO (53.73% vs 93.33%, *P* < .001), cTnT (66.42% vs 96.67%, *P* < .01), N-pro BNP (62.69% vs 90.00%, *P* < .01), CK-MB (54.48% vs 80.00%, *P* = .01), Scr (52.24% vs 73.33%, *P* = .04), BUN (64.18% vs 83.33%, *P* = .04), APTT (43.28% vs 76.67%, *P* < .01), and PT (50.75% vs 80.00%, *P* < .01) was lower than that of non-early death patients. Moreover, patients with a persistent decrease in Hb (72.39% vs 100%, *P* < .01) and a persistent decrease in PLT (41.79% vs 66.67%, *P* = .01) had a lower incidence of early death compared with non-early death patients.

APTT = activated partial thromboplastin time, BUN = blood urea nitrogen, CK-MB = creatine kinase-MB, CRP = C-reactive protein, cTnT = cardiac troponin T, D-D = D-dimer, Hb = hemoglobin, IL-6 = interleukin-6, LDH = lactate dehydrogenase, LOS = length of stay, LYM = lymphocytes, MYO = myoglobin, N-pro BNP = amino-terminal pro-brain natriuretic peptide, PCT = procalcitonin, PLT = platelets, PT = prothrombin time, RDW = red cell distribution width, Scr = serum creatinine.

* *P* < .05, the difference between patients in the 2 epidemic periods was statistically significant.

### 3.4. Influencing factors of early death

Defining “whether death occurred within 28 days after symptom onset” as the dependent variable, and taking the statistically significant difference indicators between early death and non-early death patients as independent variables for binary logistic regression analysis (the coding scheme for dummy variables – applied to all binary and multi-category predictors in the binary logistic regression analysis – is fully described in the Supplementary Table, Supplemental Digital Content 1). This analysis identified 9 independent predictors of early death (all *P* < .05): “The second epidemic period,” “With coronary heart disease,” “With cerebrovascular disease,” “New-onset anemia during hospitalization,” “0.5 × 10^9^/L ≤ valley value of LYM < 0.8 × 10^9^/L,” “250 U/L ≤ peak value of LDH < 400 U/L,” “peak value of BUN > 18 mmol/L,” “60 g/L ≤ valley value of Hb < 90 g/L,” and “LDH with continuous increase.” Among these, 5 were significant risk factors: “the second epidemic period (odds ratio [OR] = 44.76, *P* < .01),” “with coronary heart disease (OR = 38.96; *P* < .01),” “with cerebrovascular disease (OR = 24.65; *P* < .01),” “peak value of BUN > 18 mmol/L (OR = 23.58; *P* < .01),” and “LDH with continuous increase (OR = 9.86; *P* < .01).” In contrast, 4 were robust protective factors: “new-onset anemia during hospitalization (OR = 0.01; *P* < .01),” “0.5 × 10^9^/L ≤ valley value of LYM < 0.8 × 10^9^/L (OR = 0.02; *P* < .01),” “250 U/L ≤ peak value of LDH < 400 U/L (OR = 0.01; *P* < .01),” and “60 g/L ≤ valley value of Hb < 90 g/L (OR = 0.01; *P* < .01).” Notably, “the second epidemic period” exhibited the strongest association with early mortality – greater than any other covariate – in this model. Full regression estimates are presented in Table 5.

**Table 5:** Influencing factors for early mortality in patients with COVID-19.

Independent variable	*B*	SE	*P* value	OR	95.0% CI	
					Lower	Upper
The second epidemic period	3.80	1.29	<.01*	44.76	3.58	558.95
With coronary heart disease	3.66	1.11	<.01*	38.96	4.39	346.14
With cerebrovascular disease	3.20	1.12	<.01*	24.65	2.75	220.67
New-onset anemia during hospitalization	−4.59	1.22	<.01*	0.01	0.01	0.11
LYM (10^9^/L): 0.5 × 10^9^/L ≤ valley value of LYM < 0.8 × 10^9^/L	−4.00	1.38	<.01*	0.02	0.01	0.27
LDH (U/L): 250 U/L ≤ peak value of LDH < 400 U/L	−4.86	1.58	<.01*	0.01	0.01	0.17
BUN (mmol/L): peak value of BUN > 18 mmol/L	3.16	1.07	<.01*	23.58	2.87	193.81
Hb (g/L): 60 g/L ≤ valley value of Hb < 90 g/L	−4.98	1.38	<.01*	0.01	0.01	0.10
LDH with continuous increase	2.29	0.97	.02*	9.86	1.47	66.00

* *P* < .05, which indicated that these 9 independent variables above the table were influencing factors of early death. At the significance level of *P* < .05, an odds ratio (OR) >1.0 indicates a statistically significant risk factor, whereas an OR <1.0 indicates a statistically significant protective factor. Of the 9 factors examined, 5 were identified as risk factors: “The epidemic period (OR = 44.76),” “With coronary heart disease (OR = 38.96),” “With cerebrovascular disease (OR = 24.65),” “peak value of BUN > 18 mmol/L (OR = 23.58),” and “LDH with continuous increase (OR = 9.86).” The remaining 4, “The valley value of LYM: 0.5–0.8 × 10^9^/L (OR = 0.02),” “New-onset anemia during hospitalization (OR = 0.01),” “The peak value of LDH: 250 to 400 U/L (OR = 0.01),” and “The valley value of Hb: 60 to 90 g/L (OR = 0.01),” were associated with reduced odds of the outcome and thus classified as protective factors. Notably, “The epidemic period” showed the strongest association with adverse outcome (highest OR), whereas “LDH with continuous increase” showed the weakest effect among the risk factors, though still robust and statistically significant.

BUN = blood urea nitrogen, CI = confidence interval, COVID-19 = coronavirus disease 2019, Hb = hemoglobin, LDH = lactate dehydrogenase, LYM = lymphocytes, OR = odds ratio, SE = standard error.

## 4. Discussion

The demographic and clinical profiles of the 164 COVID-19-related deaths in this study aligned with the findings of numerous prior investigations.^[9–11]^ Most of the affected individuals were elderly, with 97.56% of the patients being over 60 years of age. A notable sex disparity was observed, with a male-to-female ratio of 3.69:1. Preexisting medical conditions were prevalent among the patients, with 92.68% (152 cases) having at least one type of comorbidity. The most frequently reported comorbidities included hypertension, diabetes, heart disease, respiratory disorders, cerebrovascular disease, chronic kidney disease, and malignant tumors.^[12]^ However, our study presents divergent findings from existing literature. While respiratory failure has traditionally been identified as the leading cause of death in COVID-19 cases,^[13,14]^ our data indicate that multiple organ failure was the most common direct cause of death, accounting for 46.95% (77 cases) of fatalities. Respiratory failure was the second most frequent cause, affecting 43.29% (71 cases) of patients. In comparison to the research conducted by Zhou et al,^[15]^ the median time from symptom onset to admission in our study was slightly shorter (5.00 vs 8.86 days), while the median LOS in our cohort was much longer (12.50 vs 7.55 days), and the median time from symptom to death in our study was found to be largely consistent with their findings (18.00 vs 17.37 days).

Analysis of Table 1 revealed that the primary distinction in the fundamental characteristics of patients who succumbed to COVID-19 between the first and second epidemic period lies in the “number of comorbidities.” Notably, the majority of patients who died during the second period had 3 or more concurrent underlying diseases, suggesting that this factor may have significantly influenced the propensity for early death during this period. Further insights from Table 2 indicate a higher proportion of early deaths in the second epidemic phase, with 60.87% (14 out of 23) of patients experiencing early mortality compared with 85.11% (120 out of 141) in the first phase (*P* < .01). Additionally, the time from symptom onset to death was marginally reduced in the second period, with a median duration of 21.00 days versus 18.00 days in the first phase (*P* = .01). A potential explanation for these findings is that the severity of the conditions among patients admitted during the second period was markedly higher than that observed during the initial period. This is exemplified by the fact that a significant number of patients admitted during the second period presented with respiratory distress, affecting 30.50% (43 cases) of the cohort, a condition that was not reported in the first phase.

At the outset of this study, we hypothesized that patients exhibiting more pronounced malignant progression during hospitalization would be at an increased risk of early death. However, the clinical data presented in Table 2 provide a counterintuitive finding, indicating that although patients during the first epidemic period demonstrated more pronounced malignant progression, they had a lower proportion of early deaths. The specific clinical characteristics revealed that, during the first epidemic peak, the proportion of patients with a continuous increase in CRP, PCT, and CK-MB and a continuous decrease in PLT during hospitalization were all significantly higher than those observed during the second period (*P* < .05). Furthermore, the peak values of CK-MB, LDH, APTT, PT, D-D, and RDW during the first period were significantly higher than those during the second period (*P* < .05). To reconcile this discrepancy, the researchers categorized all participants based on the occurrence of early death and conducted a subsequent analysis. However, the findings continue to challenge the initial hypothesis. The final analysis revealed that out of the 37 clinical observation indicators, differences were observed in 22 indicators between patients who experienced early death and those who did not (Table 4). Notably, only 1 indicator, that is, “the peak value of albumin,” aligned with the hypothesis, suggesting that patients with lower albumin levels were more likely to experience early death. This finding may be attributed to the fact that hypoalbuminemia can exacerbate infection and negatively impact the nutritional status of the body, thereby potentially worsening the progression of COVID-19.^[16]^

In this study, we focused on 5 key observation indicators that collectively serve as a proxy for the intensity of the cytokine storm in severe COVID-19 patients: “the peak value of CRP,” “continuous increase in CRP levels,” “continuous increase in PCT levels,” “the peak value of IL-6,” and “continuous increase in IL-6 levels.” These indicators are pivotal in assessing the dysregulation of anti-inflammatory and pro-inflammatory response systems, which are characteristic of severe COVID-19 cases. An imbalance in these systems can trigger an excessive release of inflammatory mediators, such as CRP, PCT, and IL-6. A surge in these factors can have detrimental effects on cardiac function, potentially causing myocardial cell hypertrophy or even apoptosis. This pathological process may culminate in myocardial remodeling, heart failure, and other severe outcomes, underscoring the critical role of cytokine storm management in the clinical care of COVID-19 patients.^[17]^

“The valley value of LYM” is a critical indicator of a patient’s immune status. Lymphopenia, characterized by a significant reduction in lymphocyte levels, is a notable feature observed in COVID-19 patients. This phenomenon may arise from several mechanisms, including immune consumption due to an exaggerated immune response^[18]^ or from the redistribution of LYM, where a substantial number migrate to the site of infection, leading to a decrease in peripheral lymphocyte counts. The implications of lymphopenia extend beyond its presence as a clinical sign, and it has been increasingly recognized as a potential predictor of disease severity and mortality in COVID-19. Most studies have corroborated this association, suggesting that lymphopenia may be a significant factor associated with adverse outcomes in patients with COVID-19.^[19,20]^ This highlights the importance of monitoring lymphocyte levels as part of the clinical assessment and management strategy for COVID-19 patients.

There were 4 indicators, “continuous increase in MYO, cTnT, N-pro BNP, and CK-MB levels during hospitalization,” jointly serving as a comprehensive gauge of myocardial injury. These biomarkers are pivotal in assessing the extent of cardiac muscle damage sustained by patients with COVID-19. Myocardial injury is a prevalent complication among individuals with COVID-19 and has been identified as a significant predictor of in-hospital mortality.^[21]^ Serial elevation of Scr and BUN are 2 pivotal indicators that collectively reflect the severity of kidney function damage in patients with COVID-19. These biochemical markers are integral to the assessment of renal health and are indicative of the functional capacity of the kidneys to filter waste products from the blood. Acute kidney injury (AKI) is a frequent complication in hospitalized COVID-19 patients. Extensive research has demonstrated a strong correlation between AKI development and elevated mortality rates among COVID-19 patients.^[22]^ The sustained elevation of these cardiac and renal biomarkers during hospitalization is indicative of ongoing myocardial and kidney function damage, which may contribute to the severity of the disease and the likelihood of adverse outcomes.

The trio of indicators in this study, “the peak value of PT,” and “the continuous increase in APTT and PT levels,” served as a collective reflection of the coagulation function disorder in COVID-19 patients. Severe COVID-19 has the propensity to trigger the extrinsic coagulation pathway through diverse mechanisms, resulting in coagulation abnormalities that can significantly impact patient outcomes.^[23,24]^ Additionally, the sextet of indicators, “the valley value of Hb/PLT,” “continuous decrease in Hb/PLT levels,” “new-onset anemia during hospitalization,” and “the peak value of RDW,” jointly indicated the hematological system’s disruption. Research has consistently demonstrated that anemia at the time of admission and during hospitalization in COVID-19 patients is significantly associated with an elevated mortality rate.^[25]^ Furthermore, increased RDW at admission and during hospitalization correlates with an increased mortality rate, particularly the rise in RDW during hospitalization, which is associated with a heightened risk of patient death.^[26]^ The incidence of reduced PLT in COVID-19 fatalities is alarmingly high, reaching up to 57.1%, as indicated in a previous study.^[27]^ Moreau et al further elucidated that the decline in PLT count is intimately linked to the prognosis of severe COVID-19 patients,^[28]^ underscoring the critical role of PLT function in the pathophysiology and clinical course of the disease.

Based on the findings of the aforementioned studies, it can be deduced that an escalated cytokine storm is inversely proportional to immune function and is directly correlated with the severity of myocardial and renal impairment as well as with perturbations in coagulation and hematological systems. Consequently, this leads to a more precipitous progression in patients with COVID-19, increasing their risk of premature mortality.^[29]^ By contrast, the data presented in Table 4 suggest an opposing trend, potentially attributable to the mitigating effect of medical interventions on the timing of fatal outcomes. Moreover, in cases of severe and critical illness, the absence of conspicuous symptoms or markedly abnormal clinical indicators during hospitalization may result in inadequate clinical vigilance. This oversight could preclude opportunities for timely and appropriate therapeutic intervention, thereby precipitating early death.

The regression analysis results (Table 5) identified several independent predictors of early death among hospitalized patients with severe COVID-19. Patients admitted during the second epidemic period, dominated by the Omicron variant, exhibited a markedly elevated risk of early death (OR = 44.76; 95% CI: 3.58–558.95; *P* < .01). Although Omicron is associated with lower intrinsic virulence, its clinical presentation in elderly patients often features atypical, subtle, or delayed symptom onset, particularly respiratory deterioration, leading to delayed recognition and intervention. This diagnostic inertia, rather than attenuated pathogenicity per se, likely contributes to the observed increase in early mortality. Coronary heart disease (OR = 38.96; 95% CI: 4.39–346.14; *P* < .01) and cerebrovascular disease (OR = 24.65; 95% CI: 2.75–220.67; *P* < .01) were also strong independent risk factors. These comorbidities predispose patients to myocardial injury and cerebrovascular lesions, which severe acute respiratory syndrome-coronavirus-2 infection can exacerbate via endothelial dysfunction, hypercoagulability, and systemic inflammation, establishing a self-amplifying pathological loop. Supporting this, prior evidence indicates that COVID-19 patients with concurrent myocardial injury face an approximately 26-fold increase in all-cause mortality.^[30]^ Notably, a peak BUN level exceeding 18 mmol/L conferred a 23.58-fold higher risk of early death (OR = 23.58; 95% CI: 2.87–193.81; *P* < .01), whereas moderate BUN elevation (7.5–18 mmol/L) showed no statistically significant association. This nonlinear relationship suggests a clinically meaningful threshold: BUN > 18 mmol/L reflects profound azotemia, commonly seen in multiorgan failure, AKI with high catabolic stress, or end-stage renal impairment,^[31]^ thereby serving as a red-flag biomarker for imminent physiological decompensation.

Elevated LDH reflects cumulative tissue damage across multiple organs – including lungs, myocardium, liver, and skeletal muscle. While Tambunan reported that admission LDH ≥ 256 U/L independently predicts disease severity,^[32]^ our analysis revealed that a continuous increase in LDH during hospitalization was a stronger predictor of early death (OR = 9.86; 95% CI: 1.47–66.00; *P* < .01). This dynamic pattern signaled progressive organ injury, failure of endogenous repair mechanisms, or uncontrolled inflammatory cascades. Intriguingly, a peak LDH level between 250 and 400 U/L was associated with a substantially reduced risk of early death (OR = 0.01; 95% CI: 0.01–0.17; *P* < .01), suggesting that mild-to-moderate LDH elevation may reflect a controlled, nonprogressive injury response, consistent with preserved compensatory capacity and favorable treatment responsiveness. Some studies have shown that anemia is associated with a higher in-hospital mortality rate and a higher incidence of severe disease in COVID-19 hospitalized patients.^[33]^ Contrary to expectations, new-onset anemia during hospitalization (OR = 0.01; 95% CI: 0.01–0.11; *P* < .01) and moderate baseline anemia (Hb 60–90 g/L; OR = 0.01; 95% CI: 0.01–0.10; *P* < .01) were both associated with significantly lower odds of early death. These findings imply that such hematologic changes may mark a less fulminant disease trajectory – characterized by slower clinical deterioration, retained bone marrow reserve, or effective adaptation to hypoxic stress – rather than irreversible exhaustion. Similarly, patients with moderate LYM (LYM count 0.5–0.8 × 10^9^/L) exhibited a 98% lower risk of early death compared with those maintaining counts ≥ 1.0 × 10^9^/L (OR = 0.02; 95% CI: 0.01–0.27; *P* < .01). Although a reduced LYM count is highly correlated with the severity of the patient’s condition,^[34]^ this counterintuitive association in this article underscores the nonlinear immunobiology of severe COVID-19: moderate lymphopenia may represent a balanced immune state, neither hyperactivated (avoiding cytokine storm) nor profoundly suppressed (preserving antiviral surveillance), thereby enabling effective viral control without lethal immunopathology.

Collectively, this study advances understanding of early mortality determinants across 2 pivotal phases of China’s pandemic response. It identifies Omicron-dominant epidemiology, cardiovascular and cerebrovascular comorbidities, BUN > 18 mmol/L, and rising LDH as robust, independent risk factors for early death. Conversely, moderate lymphopenia, modest LDH elevation (250–400 U/L), moderate anemia, and new-onset anemia appear to reflect adaptive physiological responses, rather than decompensation, suggesting a window for timely therapeutic intervention to mitigate early mortality risk. These insights hold direct relevance for risk stratification, resource allocation, and personalized management of critically ill COVID-19 patients. This study has several limitations. First, its single-center design and modest sample size constrain generalizability. Critically ill patients constituted the majority of admissions at the participating hospital, resulting in a higher observed mortality rate and potential selection bias toward severe disease phenotypes. Second, exposure classification – based solely on epidemic period timing (first vs second period) and binary early death status – was applied post hoc and lacked granularity; it did not account for key clinical confounders such as treatment protocols (e.g., corticosteroid dosing, antiviral timing), specific pharmacotherapies (e.g., IL-6 inhibitors, anticoagulation regimens), or vaccination history, all of which significantly modulate COVID-19 outcomes. Consequently, residual confounding likely attenuated or biased effect estimates. Third, while the analysis captured directional trends in serial biomarkers (e.g., rising LDH, peaking BUN), it employed static thresholds or categorical summaries rather than modeling their time-varying trajectories. As a result, the study could not quantify how the rate, timing, or magnitude of biochemical change dynamically influenced early survival probability. Future work should prioritize prospective, multicenter collaboration to increase statistical power and clinical diversity. Detailed longitudinal collection of treatment variables, vaccination records, and high-frequency biomarker measurements would enable application of time-dependent Cox proportional hazards models, thereby yielding more precise, clinically actionable insights into the temporal dynamics of mortality risk.

## Acknowledgments

The authors would like to thank the Department of Information of the General Hospital for their contributions to the data collection.

## Author contributions

**Conceptualization:** Jing-mei Ding, Jin-bing Du.

**Resources:** Min Li, Meng-yu Shen.

**Validation:** Min Li, Meng-yu Shen.

**Formal analysis:** Lei Han

**Investigation:** Lei Han.

**Writing – review & editing:** Min Li, Meng-yu Shen.


