# Supplementary material for: Analysis of the clinical characteristics of patients with COVID-19-related deaths during the two epidemic periods in a tertiary hospital in Wuhan, China: A retrospective study
Source: Medicine (Baltimore). 2026 Jun 26;105(26):e49397. doi: 10.1097/MD.0000000000049397 (PMC13313744; doi:10.1097/MD.0000000000049397)
Supplement: Supplementary file 1 [file medi-105-e49397-s001.docx]

**Supplementary table**

**The settings of dummy variables in binary logistic regression**

| Independent variable | Control group | Dummy variables |
| --- | --- | --- |
| The epidemic period | The first epidemic period | The second epidemic period |
| Gender | Male | Female |
| Age | <60岁 | ≥60岁 |
| Smoking | No | Yes |
| Alcohol | No | Yes |
| With coronary heart disease | No | Yes |
| With diabetes | No | Yes |
| With cerebrovascular disease | No | Yes |
| With chronic renal insufficiency | No | Yes |
| With malignancy | No | Yes |
| With hyperlipidemia | No | Yes |
| With COPD | No | Yes |
| With senile dementia | No | Yes |
| With bronchiectasis | No | Yes |
| Number of comorbidities | zero | One |
|  |  | Two |
|  |  | Three or more |
| Peak value of CRP (mg/L) | ≤10mg/L | 10 mg/L < Peak value of CRP≤50 mg/L |
|  |  | 50 mg/L < Peak value of CRP≤100 mg/L |
|  |  | 100 mg/L < Peak value of CRP≤200 mg/L |
|  |  | Peak value of CRP >200 mg/L |
| CRP with continuous increase | No | Yes |
| Peak value of PCT (ng/ml) | ≤0.5ng/ml | 0.5 ng/ml < Peak value of PCT≤2.0 ng/ml |
|  |  | 2.0ng/ml< Peak value of PCT≤10.0 ng/ml |
|  |  | Peak value of PCT>10 ng/ml |
| PCT with continuous increase | No | Yes |
| Peak value of IL-6（pg/ml） | ≤7.0pg/ml | 7.0pg/ml< Peak value of IL-6≤150.0 pg/ml |
|  |  | 150.0pg/ml< Peak value of IL-6≤500.0 pg/ml |
|  |  | Peak value of IL-6>500.0 pg/ml |
| IL-6 with continuous increase | No | Yes |
| Valley value of LYM (10^9^/L) | ≥1.0*10^9^/L | 0.8*10^9^/L≤ Valley value of LYM<1.0*10^9^/L |
|  |  | 0.5*10^9^/L≤ Valley value of LYM<0.8*10^9^/L |
|  |  | 0.2*10^9^/L≤ Valley value of LYM<0.5*10^9^/L |
|  |  | Valley value of LYM<0.2*10^9^/L |
| LYM with continuous decrease | No | Yes |
| Hyperkalemia occurrence | No | Yes |
| Peak value of MYO（ng/ml） | <200ng/ml | 200 ng/ml≤ Peak value of MYO<500 ng/ml |
|  |  | 500 ng/ml≤ Peak value of MYO<1500 ng/ml |
|  |  | 1500 ng/ml≤ Peak value of MYO<3000 ng/ml |
|  |  | Peak value of MYO≥3000 ng/ml |
| MYO with continuous increase | No | Yes |
| Peak value of cTnT（ng/ml） | ≤0.05ng/ml | 0.05ng/ml< Peak value of cTnT ≤0.5 ng/ml |
|  |  | 0.5ng/ml< Peak value of cTnT ≤2.0 ng/ml |
|  |  | Peak value of cTnT>2.0ng/ml |
| cTnT with continuous increase | No | Yes |
| Peak value of N-pro BNP (pg/ml) | <5000pg/ml | 5000pg/ml ≤Peak value of N-pro BNP <10000 pg/ml |
|  |  | 10000pg/ml ≤Peak value of N-pro BNP <35000 pg/ml |
|  |  | Peak value of N-pro BNP≥35000 pg/ml |
| N-pro BNP with continuous increase | No | Yes |
| Peak value of CK-MB（ng/ml） | <25 ng/ml | 25 ng/ml≤ Peak value of CK-MB<100 ng/ml |
|  |  | 100 ng/ml≤ Peak value of CK-MB<300 ng/ml |
|  |  | 300 ng/ml≤ Peak value of CK-MB<500 ng/ml |
|  |  | Peak value of CK-MB≥500 ng/ml |
| CK-MB with continuous increase | No | Yes |
| Valley value of albumin (g/L) | ≥35g/L | 30g/L≤ Valley value of albumin<35 g/L |
|  |  | 20g/L≤ Valley value of albumin<30 g/L |
|  |  | 20g/L≤ Valley value of albumin<25 g/L |
|  |  | Valley value of albumin<20 g/L |
| Albumin with continuous decrease | No | Yes |
| Peak value of LDH (U/L) | <250U/L | 250U/L≤ Peak value of LDH <400U/L |
|  |  | 400U/L≤ Peak value of LDH <800U/L |
|  |  | 800U/L≤ Peak value of LDH <1000U/L |
|  |  | Peak value of LDH≥1000U/L |
| LDH with continuous increase | No | Yes |
| Peak value of Scr (umol/L) | ≤110umol/L | 110umol/L< Peak value of Scr ≤177umol/L |
|  |  | 177umol/L< Peak value of Scr ≤354umol/L |
|  |  | 354umol/L< Peak value of Scr ≤500umol/L |
|  |  | Peak value of Scr >500 umol/L |
| Scr with continuous increase | No | Yes |
| Peak value of BUN (mmol/L) | ≤7.5mmol/L | 7.5mmol/L < Peak value of BUN≤ 12.5 mmol/L |
|  |  | 12.5mmol/L < Peak value of BUN≤ 18 mmol/L |
|  |  | Peak value of BUN> 18 mmol/L |
| BUN with continuous increase | No | Yes |
| Peak value of APTT (s) | ≤36s | 36s < Peak value of APTT≤ 50s |
|  |  | 50s < Peak value of APTT≤ 70s |
|  |  | 70s < Peak value of APTT≤ 100s |
|  |  | Peak value of APTT>100s |
| APTT with continuous increase | No | Yes |
| Peak value of PT (s) | ≤12s | 12s <Peak value of PT≤ 14s |
|  |  | 14s <Peak value of PT≤ 16s |
|  |  | Peak value of PT>16s |
| PT with continuous increase | No | Yes |
| Peak value of D-D(mg/L) | ≤0.5ug/ml | 0.5ug/ml< Peak value of D-D≤ 1.0 ug/ml |
|  |  | 1.0ug/ml< Peak value of D-D≤ 3.0 ug/ml |
|  |  | 3.0ug/ml< Peak value of D-D≤10.0 ug/ml |
|  |  | Peak value of D-D> 10.0 ug/ml |
| D-D with continuous increase | No | Yes |
| Valley value of Hb (g/L) | ≥110g/L | 90g/L≤ Valley value of Hb <110g/L |
|  |  | 60g/L≤ Valley value of Hb <90g/L |
|  |  | Valley value of Hb <60g/L |
| Hb with continuous decrease | No | Yes |
| New-onset anemia during hospitalization | No | Yes |
| Valley value of PLT (10^9^/L) | ≥100*10^9^/L | 50*10^9^/L≤ Valley value of PLT< 100*10^9^/L |
|  |  | 20*10^9^/L≤ Valley value of PLT< 50*10^9^/L |
|  |  | Valley value of PLT< 20*10^9^/L |
| PLT with continuous decrease | No | Yes |
| Peak value of RDW (%) | ≤14.5% | 14.5%< Peak value of RDW ≤15.5% |
|  |  | 15.5%< Peak value of RDW ≤17.0% |
|  |  | Peak value of RDW >17.0% |
